# Prognostic factors for return to work in patients affected by chronic low back pain: a systematic review

**DOI:** 10.1007/s12306-024-00828-y

**Published:** 2024-06-12

**Authors:** F. Russo, G. F. Papalia, L. A. Diaz Balzani, G. Stelitano, B. Zampogna, L. Fontana, G. Vadalà, S. Iavicoli, R. Papalia, V. Denaro

**Affiliations:** 1 Operative Research Unit of Orthopaedic and Trauma Surgery, Department of Orthopaedic and Trauma Surgery, Fondazione Policlinico Universitario Campus Bio-Medico, Via Alvaro del Portillo 200, 00128 Rome, Italy; 2 Research Unit of Orthopaedic and Trauma Surgery, Department of Medicine and Surgery, Università Campus Bio-Medico di Roma, Via Alvaro del Portillo, 00128 Rome, Italy; 3 Occupational Health Unit, Department of Public Health, Federico II University of Naples, Naples, Italy; 4 Directorate for Communication and International Affairs, Ministry of Health, Rome, Italy

**Keywords:** Low back pain, Return to work, Sick leave, Prognostic factors, Systematic review

## Abstract

Chronic low back pain (LBP) represents a leading cause of absenteeism from work. An accurate knowledge of complex interactions is essential in understanding the difficulties of return to work (RTW) experienced by workers affected by chronic LBP. This study aims to identify factors related to chronic LBP, the worker, and the psycho-social environment that could predict and influence the duration of an episode of sick leave due to chronic LBP.Studies reporting the relation between prognostic factors and absenteeism from work in patients with LBP were included. The selected studies were grouped by prognostic factors. The results were measured in absolute terms, relative terms, survival curve, or duration of sick leave. The level of evidence was defined by examining the quality and the appropriateness of findings across studies in terms of significance and direction of relationship for each prognostic factor.A total of 20 studies were included. Prognostic factors were classified in clinical, psycho-social, and social workplace, reaching a total of 31 constructs. Global conditions with less favorable repercussions on worker’s lives resulted in a delay in time to RTW. Older age, female, higher pain or disability, depression, higher physical work demands, and abuse of smoke and alcohol have shown strong level of evidence for negative outcomes.High global health well-being, great socioeconomic status, and good mental health conditions are decisive in RTW outcomes. Interventions that aim at RTW of employee’s sick-listed with LBP should focus on psycho-social aspects, health behaviors, and workplace characteristics.

## Introduction

Low back pain (LBP) is considered a global public health problem, representing one of the most causes of absenteeism from work. In particular, the annual productivity losses from missed workdays due to LBP are estimated at $28 billion in the USA alone, and this condition currently represents the main cause of disability, affecting nearly 600 million workers worldwide [1]. LBP is the third most common cause of years lived with disability (YLDs) worldwide, accounting for nearly 10% of all YLDs [2]. Despite developments in objective health measures, time of absenteeism from work due to LBP has also shown an increase in European countries [3, 4]. Reasons underlying this situation include degree of disease severity, different types of treatments, compliance with the interventions, but also lifestyle risk factors and characteristics of the patient’s work activities. In detail, the occupational risk factors that may be involved in the etiopathogenesis of LBP are numerous and include manual handling of heavy loads, awkward and prolonged postures, whole-body mechanical vibrations, and work-related stress [5]. Therefore, when the work activities required by the specific job carried out by the patient with LBP involve exposure to these occupational risk factors, an increase in absenteeism and a delayed return to work (RTW) often occur [6]. Consequently, an accurate and well-rounded knowledge of the complex interactions between workers health conditions, psychosocial factors and workplace issues is essential in understanding the difficulties of returning to work experienced by workers affected by chronic LBP [7]. Indeed, RTW represents a multifactorial process influenced by physical, psychological, and social factors, and provides a reliable description of work success and socioeconomic status worldwide [8]. Subjects unable to RTW because of chronic injury or illness can experience greater physical ailments and poorer psycho-social adjustment (increased anxiety, depression, social isolation) [9, 10]. Current literature data widely show that recovery beliefs, pain-related behaviors, work-related risk factors, and health-related conditions must be assessed as potential influencers of RTW [11–13]. In this regard, several studies have tried to identify risk factors for absenteeism from work correlated to LBP [14, 15]. However, their findings do not allow to obtain definitive conclusions on elements or parameters to considered to achieve an early RTW of workers with LBP. In fact, most observational studies focusing on pain and RTW have mainly paid attention to acute LBP [16]. The aim of this study is to identify factors related to LBP, the worker, the job, and the psycho-social environment, that could predict and influence the duration of an episode of sick leave and time away from work due to chronic LBP.

## Materials and methods

### Definition of prognostic factors and outcomes

Low back pain must be considered a multifactorial problem. Individual, psycho-social, and work-related factors seem to influence its onset, duration, and outcome. Several predictive factors have been identified, including every aspect of personal life, job and workplace, psychological environment, and specific low back pain characteristics of patients. Therefore, this review focused on the time away from work, defined both as “sick leave” and “return to work”.

### Literature review

This review was assessed in accordance with the Preferred Reporting Items for Systematic Reviews and Meta-Analysis (PRISMA) guidelines. The search strategy was conducted on PubMed, MEDLINE, Cochrane, and Scopus databases. The following string was used: (((“low back pain”[MeSH Terms] OR (“low”[All Fields] AND “back”[All Fields] AND “pain”[All Fields]) OR “low back pain”[All Fields]) AND (“return to work”[MeSH Terms] OR (“return”[All Fields] AND “work”[All Fields]) OR “return to work”[All Fields])) OR (“low back pain”[MeSH Terms] OR (“low”[All Fields] AND “back”[All Fields] AND “pain”[All Fields]) OR “low back pain”[All Fields])) AND (“sick leave”[MeSH Terms] OR (“sick”[All Fields] AND “leave”[All Fields]) OR “sick leave”[All Fields]). The reviewers conducted the last investigation on 31st May 2023. Two autonomous researchers (G.S. and F.R.) independently performed the search. Every study has initially been chosen by utilizing title and abstract, and duplicate studies were removed. Then, they consulted the full-text article and performed an accurate reading of every chosen research, obtaining data to reduce selection bias. A cross-reference exploration of the studies was made to get additional related research. Every study describing any prognostic factors related to absenteeism from work due to LBP was considered.

### Inclusion criteria

Studies published from 2012 to May 2023 involving patients with an episode of LBP and sick leave, with a duration of more than 12 weeks, reporting the relation between at least one prognostic factor and absenteeism to work were included. Moreover, the results had to be measured in absolute terms (rate), relative terms (odds ratio, rate ratio, hazard ratio), survival curve, or duration of sick leave. Studies antecedents to 2012, systematic reviews and studies concerning the acute and subacute LBP were excluded.

### Quality assessment

Two independent investigators (G.S. and F.R.) scored the quality of included studies. Studies evaluation was performed by the quality assessment list composed of 17 items, organized into three categories: methodological quality, quality of measurement of prognostic factors, and statistical quality [17]. Items were the following: adequate description of the study population, description of response, the extent and length of follow-up, an explicit definition of time to RTW, the number of prognostic factors measured, and the quality of data presentation. The maximum score for all items was 17. Studies were at high quality (range 12–17 points), moderate quality (range 9–11 points), or low quality ( < 9 points).

### Data extraction

Information concerning the definition of prognostic factor, outcomes, country, setting, association estimate, and sample size were extracted from each article. For the risk of no RTW, a ratio larger than one was considered significative of delay in time until RTW.

### Level of evidence

The selected studies were grouped by prognostic factors. The level of evidence was defined by examining the quality of each study and the appropriateness of findings across studies in terms of significance and direction of relationship for each prognostic factor. In particular, the level of evidence has been identified by the following criteria:

- Strong evidence when prognostic factors appeared in multiple high-quality studies.
- Moderate evidence when prognostic factors appeared in one high-quality study and one or lower-quality studies or multiple lower-quality studies.
- Insufficient evidence when prognostic factors appeared in only one study available or several studies with inconsistent findings.

## Results

### Literature search

**Fig. 1:**
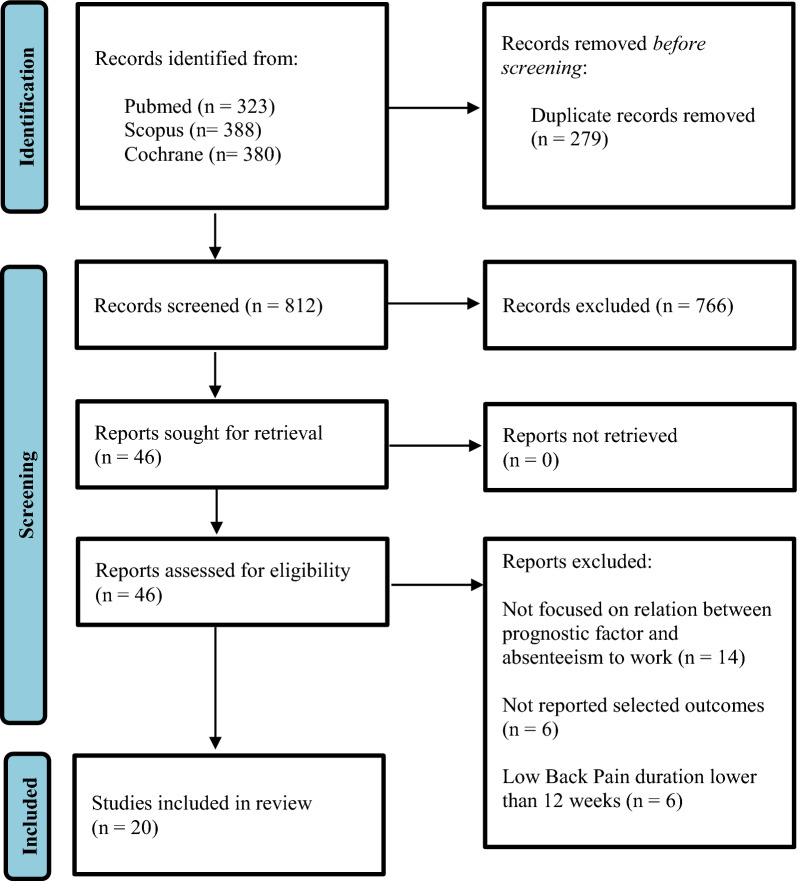
Preferred reporting items for systematic review and meta-analysis (PRISMA 2020)

From the initial research, 1091 articles were obtained, of which 279 were removed as duplicates. After screening all titles and abstracts, 46 articles were identified as suitable for a more full-text review. Finally, 20 publications satisfied all the inclusion criteria (Fig. 1). Studies characteristics are reported in Table 1.

**Table 1:** Characteristics of included studies

Author	Year	Sample size	Setting	Outcomes	N factors	Results
Opsahl et al. [18]	2016	574	Employees (data extraction from CINS trial)	RTW	2 (Job satisfaction; expectance of RTW) + Covariates (fear avoidance beliefs; subjective health complaints; disability; co-worker social support; emotional distress; age; sex; smokers; education, sociodemographic factors)	High expectancies were a strong and significative predictors factors of RTW; high level of job satisfaction was not a significative predictor; men had higher odds of RTW compared to women; high perceived co-worker social support, being a non-smoker and not reporting emotional distress contributed significantly to prediction RTW for men compared to women.
Besen et al. [19]	2015	241	Employers referred to the clinics, primary care providers, emergency rooms (sample drawn from a larger study of 496 adult seeking treatment for work-related LBP)	Day of absence	7 (Pain, catastrophizing, fear avoidance beliefs, organizational support, co-worker support, RTW confidence, RTW expectations)	For day of absence, significant correlations were observed with all predictors except organizational support and co-worker support.
Jensen et al. [20]	2013	442	Patients recruited from Research Unit of the Spine center (Denmark)	Unsuccessful-RTW	2 clinical predictor factors (Pain, side-flexion) + 5 psychosocial predictor factors (bodily distress, low expectations of RTW, blaming the work for pain, no home ownership, drinking alcohol less than once/month)	Both clinical and psychosocial predictors seemed to contribute to the risk for Unsuccessful-RTW.
Norbye et al. [21]	2016	58	Patients from the multidisciplinary clinics for LBP at the Department of Neurology, Molde Hospital	RTW	Early intervention	Early intervention directly compared with an ordinary waiting list did not significantly affect the RTW.
Macias-Toronjo et al. [22]	2020	129	Patients from occupational health clinic	Duration of sickness leave	4 Clinical Variables (Kinesiophobia, catastrophizing, disability, pain) + 4 Sociodemographic variables (age, sex, occupational level, educational level)	Educational level, occupational level (sociodemographic variables) and kinesiophobia (clinical variable) were associated with sickness leave; Kinesiophobia and catastrophizing were associated with duration of sickness leave, while no one sociodemographic variables was associated.
Leung et al. [23]	2021	158	Patient recruited from a tertiary health center	RTW	5 Categorical parameters (Gender, job demand level, smoking, education, IOD) + 10 continuous parameters (age, pre-program sitting tolerance, standing tolerance, walking tolerance, VAS at rest, VAS on exertion, ODI, BDI, SFSS, BABS)	Job demand level, smoking, IOD, pre-program BDI, SFSS, and BABS were significant factor for RTW.
Carregaro et al. [24]	2020	N.R.	Patient recruited from National database (worker with LBP)	Day of absence	4 Categories: gender, type of benefits (work related and non-work related sickness benefit), economic activity (transports, rural work, public servant, industry, commerce), age	All categories are related to absence of work. In particular, men and rural activity are related to greater absence from work and in addition, both age ant type of benefit (with small difference between work and non-work related sickness benefits) significantly contribute to the absenteeism.
Lardon et al. [25]	2018	100	Patients enrolled by university outpatient clinic and university employers through international advertisement	Duration of sickness leave	9 variables: Clinical pain, LBP duration (months), pain perception threshold, tolerance threshold, pain inhibition during HNCS, pain inhibition during recovery, hypervigilance, work satisfaction, risk of poor clinical outcome	Hypervigilance, risk of poor clinical outcome and work satisfaction had shown significant correlation with LPB sick leave, despite the significance disappears when the parameters had adjusted for age, gender, type of work and intervention
Trinderup et al. [26]	2018	559	Patients referred from general practitioners, rheumatologist, municipal sickness benefit office for treatment of persistent LBP	Duration of sickness leave	24 Variables: age, sex, BMI, education, smoking, alcohol intake, leisure physical activity level, sick leave due to LBP, duration of sick leave, job status, current compensation case, physical job demands, general health status, mental health, anxiety, depression, pain intensity, disability, fear avoidance beliefs work, fear avoidance beliefs physical activity, family history of LBP, age at first episode of LBP, intervention, employment	High fear avoidance beliefs about work and being a smoker at baseline were significantly associated with unsuccessful outcome on sick leave.
Macias-Toronjo et al. [27]	2020	88	Individuals who presented to an occupational health clinic with diagnosis of non-specific LBP	Duration of sickness leave	6 Clinical variables: Kinesiophobia, catastrophizing, fear avoidance in global dimension, fear avoidance in work dimension, fear avoidance in physical activity, pain intensity + 3 Sociodemographic variables: sex, education, occupational level	Educational and occupational level appeared to be related to LBP sickness absence variable; as regard the duration of sickness absence, none of the sociodemographic variables are related to it; while all of the clinical variables show individual correlation with duration of sickness absence but none for sickness absence status.
Koopman et al. [28]	2004	51	Participants were selected by occupational physicians or medical advisors of an insurance company	RTW	4 baseline variables: sex, age, reinterpretation of pain sensations, functional disability pre-treatment + 1 variable of change: trunk flexibility	Men, younger participants, participants with lower functional disability, and participants who were using the coping strategy of reinterpretation of pain sensations more often had a higher chance of work resumption. Participants who showed a large increase in trunk flexibility during the program had a higher chance of work resumption.
Gauthier et al. [29]	2006	255	Workers who sustained a soft tissue injury to the back and participated in a community-based secondary prevention program	RTW	3 demographic variables: sex, age, work absence + 4 risk factors measures: Pain catastrophizing, Fear of movement/reinjury, Pain intensity, Depression	Sex and duration of work absence contributed significantly to the prediction of RTW. Risk factor measures contributed significantly to the prediction of return to work. Pain catastrophizing and pain severity at pre-treatment contributed significant unique variance in the prediction of RTW.
Vendrig et al. [30]	1999	147	Patients referrals to The Netherlands Back Advice Center, a network of multidisciplinary assessment and intervention centers with the work reintegration of spinal disorder patients	RTW	Demographic and socioeconomic data: age, sex, education, pain duration, disability time, number of spinal operations; physical measures: lumbar functioning, cardiovascular fitness; psychological measures: VAS, pain drawing, disability, hypochondriasis, depression, distress	About demographic and socioeconomic data, disability time and number of previous spinal surgeries appeared to be significant (negative) predictors of RT. None of the physical variables predicted RTW. About the psychological variables, hypochondriasis and distress variables predicted RTW.
Okurowski et al. [31]	2006	986	Cases of uncomplicated occupational LBP referred to NCMs were selected by National Council on Compensation Insurance part of body/nature of injury codes for uncomplicated/ noncatastrophic LBP	RTW	23 independent variables: age, appropriateness of treatment, assessment period, attorney involvement, average weekly wage, comorbidity, compliance with treatment, current medications, education level, functional capacity level, gender, intensity or duration of treatment, job demand level, language barriers, marital status, modified duty available, months on job, presentation of symptoms, prior injuries or prolonged work absences, severity of work- related injury, timeliness of referral, and workplace issues	Older age, language barriers, earlier referral to NCMs, and negative or neutral attorney attitude toward RTW were found to be significantly associated with being out of work at 6 months.
Van der Giezen et al. [32]	2000	328	Patients from a longitudinal Dutch study of sick- listed employees, part of an international comparative cohort study `Work Incapacity and Reintegration’ of LBP- patients in the private sector	RTW	5 variables: health status, history of LBP, occupational variables, job characteristics and social economic variables	RTW was independently predicted by having a better general health status, having better job satisfaction, being a bread winner, having a lower age, and reporting less pain all measured at cohort entry. This study shows that psycho-social aspects of health and work in combination with economic aspects have a significantly larger impact on RTW when compared to relatively more physical aspects of disability and physical requirements of the job.
Hansson et al. [33]	2000	2080	Patient selected from employed who had been sick-listed for a minimum of 90 days because of LBP, recruited in Denmark, Germany, Israel, Sweden, the Netherlands, and the United States	RTW	6 health measures: patient functional limitations, general health, pain intensity, vitality, social function, mental health; other variables: age, sex, treatment before sick-listing; psychological demand, decision latitude, physical job demands, surgery during the first years	About health measures: Lower pain intensity, patient functional limitations, better social functioning and better mental health, all predicted work resumption. About other variables: lower age, male gender, no treatment for the present LBP problems before sick-listing, lower psychological demands, higher decision latitude, lower physical demands, history of back surgery resulted variables with positive prediction for work resumption.
Anema et al. [34]	2004	1631	A multinational cohort of compensation claimants off work for 3–4 months due to LBP was recruited in Denmark, Germany, Israel, the Netherlands, Sweden, and the United States	RTW	Patient characteristics: gender, country, age, education and quetelet index; health-related characteristics : general health, active coping, passive coping, co-morbidity, pain intensity, sciatic pain, sick leave history due to lbp, patient functional limitations; job characteristic: working hours, job duration, firm company size, work ability, attitude toward work, physical job demands, social support, job strain; medical interventions: surgery, pain medication, passive treatment, exercise therapy, back school; work interventions: adaptation workplace, job redesign, working hours adaptation, therapeutic work resumption, job training, sheltered workshop	Adaptation of the workplace was effective on RTW rate with an adjusted hazard ratio (HR) of 1.47. Adaptation of job tasks and adaptation of working hours were effective on RTW after a period of more than 200 days of sick leave with an adjusted HR of 1.78 and 1.41 respectively. Ergonomic interventions are effective on RTW of workers long term sick listed due to LBP.
Anema et al. [35]	2009	2825	A multinational cohort of compensation claimants off work for 3–4 months due to LBP was recruited in Denmark, Germany, Israel, the Netherlands, Sweden, and the United States.	RTW	Patient characteristics: gender, country, age, education and quetelet index; health-related characteristics: general health, active coping, passive coping, co-morbidity, pain intensity, sciatic pain, sick leave history due to lbp, patient functional limitations; job characteristic: working hours, job duration, firm company size, work ability, attitude towards work, physical job demands, social support, job strain; medical interventions: surgery, pain medication, passive treatment, exercise therapy, back school; work interventions: adaptation workplace, job redesign, working hours adaptation, therapeutic work resumption, job training, sheltered workshop	About health-related variables, there was an association with earlier sustainable RTW: no co-morbidity interference, a lower pain intensity and less functional limitations. The following job characteristics were associated with earlier sustainable RTW: longer tenure, higher work ability score, less physical job demands and less job strain. There was an association with earlier sustainable RTW for the following medical interventions: surgery, pain medication, exercise therapy. Four work interventions were related to earlier sustainable RTW: adaptation of the workplace, job redesign, working hours adaptation, and therapeutic work resumption.
Gatchel et al. [36]	1994	152	Patients admitted to a 3 week functional restoration treatment program for whom l-year follow-up data could be collected	RTW	Demographic characteristics: age, sex, number of months since injury, workers compensation case, years of education; Axix I personality disorders, Axix II personality disorders	None demographic characteristics had resulted predictive for RTW. Neither type nor degree of psychopathology were significantly predictive of a patient’s ability to successfully RTW.
Gross et al. [37]	2004	114	Workers compensation claimants undergoing functional capacity evaluations following work-related low back injury	RTW	Functional capacity Evaluation	Indicators of better performance on the functional capacity evaluation were weakly associated with faster recovery.

*N* number; *CINS* cognitive interventions and nutritional supplements; *RTW* return to work; *LBP* low back pain; *IOD* injury on duty; *VAS* visual analog scale; *ODI* oswestry disability index; *BDI* beck depression inventory; *SFSS* spinal function sort score *BABS* bradburn affect balance scale; *BMI* body mass index; *NCM* nurse case manager.

### Quality assessment

The mean quality score was 11.8, ranging from 7 to 15 (Table 2). The quality of thirteen studies (72%) was high, while three (17%) studies were of moderate quality, and two (11%) were of low quality.

**Table 2:** Quality assessment of all studies

Author	A	B	C	D	E	F	G	H	I	J	K	L	M	N	O	P	Q	Overall rating
Macias-Toronjo (12/2020)	+	+	+	+	+	+	+	+	−	−	+	+	?	−	+	+	−	12
Trinderup	+	+	+	−	+	+	+	+	+	+	+	−	+	−	+	+	+	14
Lardon	+	+	−	−	−	−	+	+	−	−	+	+	+	+	+	+	+	11
Carregaro	+	+	?	−	+	−	−	−	−	−	+	+	?	?	+	+	−	7
Leung	+	+	?	−	+	+	+	+	−	−	+	+	?	+	+	+	+	12
Macias-Toronjo (08/2020)	+	+	+	+	+	+	+	+	−	−	+	+	?	−	+	+	−	12
Besen	+	+	−	+	−	−	+	+	−	+	+	+	−	+	+	+	+	12
Opsahl	+	+	+	+	−	+	+	+	?	+	+	+	−	−	+	+	+	13
Okurowski	+	+	−	+	+	+	+	+	−	+	+	+	−	−	+	+	+	13
Koopman	+	+	−	+	−	−	?	?	−	−	+	+	+	−	+	+	−	8
Gauthier	+	+	?	+	−	−	+	+	−	+	+	+	+	+	+	+	−	12
Anema (2004)	+	+	−	+	+	+	+	+	−	+	+	+	+	−	+	+	+	14
Anema (2009)	+	+	−	+	+	+	+	+	−	+	+	+	+	−	+	+	+	14
Gatchel	+	+	?	+	−	−	+	+	−	?	+	+	−	−	+	+	−	9
Gross	+	+	?	+	+	+	−	−	−	+	+	+	+	+	+	+	−	12
Van der Giezen	+	+	+	?	+	+	+	+	+	+	+	+	+	−	+	+	+	15
Vendrig	+	+	+	+	−	−	+	+	−	+	+	+	−	−	+	+	?	11
Hansson	+	+	?	+	+	+	+	+	−	−	+	+	+	−	+	+	−	12

### Evidence on prognostic factors

The identified prognostic factors were classified into three categories: clinical, psycho-social, and social workplace. Each category included factors or tools measuring the same or very similar findings, reaching a total of 31 constructs. The level of evidence for each prognostic factor is reported in Table 3.

**Table 3:** Prognostic factors evidence table

Prognostic factor	Evidence	Level of evidence
*Clinical prognostic factors*		
Sex	4H 2L	Positive—high
Age	4H 2L	Positive—high
Comorbidity	4H	Negative—high
Diagnosis	1H	Negative—moderate
Pain intensity	7H	Negative—high
Functional status	5H 1L	Positive—high
Intervention	5H 1L	Negative—high
Health	1H	Positive—moderate
Lifestyle	1H	Positive—moderate
Smoker	4H	Negative—high
Alcohol	2H	Negative—high
*Psychosocial prognostic factor*		
Expectation	3H	Positive—high
Fear avoidance	5H	Positive—high
Kinesiophobia	3H	Positive—high
Pain catastrophizing	6H	Positive—high
Cognitive appraisal	1H	Positive—moderate
Coping	1H 1L	Positive—high
Distress	4H	Negative—high
Depression	3H	Negative—high
Mental health	6H	Positive—high
Disability	2H 1L	Negative—high
Education	3H	Positive—high
*Social workplace prognostic factor*		
Socio-economic status	2H	Positive—high
Physical demands	6H 1L	Positive—high
Work-related benefits	2H 1L	Positive—high
Modify duties	2H	Positive—high
Co-worker support	2H	Positive—high
Social support	2H	Positive—high
Job satisfaction	2H	Positive—high
Attorney involvement	3H	Positive—high
Workers compensation	1H	Positive—moderate

### Results on clinical prognostic factors

There is evidence of a positive association between male sex and RTW from 4 high-quality and 2 low-quality studies.

There is high evidence of a positive association between younger and RTW from 4 high-quality and 2 low-quality studies.

Diagnosis has a moderate level of evidence, showing a significant association with RTW only in one high-level study. There is high evidence of the association between comorbidities and RTW, resulting from 4 high-quality studies. In particular, the more diseases a patient suffers from, the greater the risk of delaying the return to work.

Radiating pain was studied in none of the chosen studies. There is high evidence that pain intensity has a negative association with RTW from 7 high-quality studies. Functional status shows a high level of evidence in its association with RTW, resulting in statistically significant in 5 high-quality studies and one low quality.

A delay in referral to intervention showed a strong association with a delay in RTW. This high level of evidence was confirmed by 5 high-quality studies and one lower quality study. Only in one low-quality study the early intervention directly compared with an ordinary waiting list did not significantly affect the RTW.

Both health and lifestyle related clinical factors have a moderate level of evidence measures on RTW from only one high-quality study. Notably, being a smoker and exceeding in alcohol assumption have shown a strong association with a delay RTW, respectively, from 4 and 2 high-quality studies.

### Results on personal psycho-social factors

Researchers from three studies reported differently on the one-item question from the workability index that investigates expectations of RTW. These studies have shown a strong level of evidence for the high recovery expectation and RTW.

Pain catastrophizing, fear avoidance, coping, and kinesiophobia presented all a high level of evidence on RTW. Pain catastrophizing and fear-avoidance have been considered in most of the studies, resulting in a positive association with RTW respectively in 6 and 5 high-quality articles. Also, kinesiophobia and coping resulted in a positive statistically significant association with RTW, despite being measured in a limited number of studies. Only one high-quality study and one low-quality study reported a positive association for coping and 3 high-quality studies for kinesiophobia.

Six high-quality studies assessed the association between mental health and RTW, resulting in a strong level of positive evidence between high mental health status and an early RTW. At the opposite side, distress, depression, and disability have shown a negative relationship with RTW. Notably, these results confirm that global mental well-being has an essential influence on the time of absenteeism from work.

In only one high-quality study, cognitive appraisal has shown an association with RTW, resulting in a moderate level of evidence. Three high-quality studies found a strong correlation between disability education and RTW.

### Results on social workplace prognostic factors

Each of the social workplace prognostic factors analyzed has revealed a high level of evidence on RTW. All these factors are reported in a single category to underlying the strong correlation among each of them. Workers compensation has been evaluated in one high-quality study, showing a moderate level of evidence.

## Discussion

This study aims to identify factors that predict the duration of time away from work at the chronic stage of LBP. The factors able to influence RTW are related to LBP, worker, and job characteristics, as well as to the psycho-social environment. Accurate comprehension of the risk factors related to absenteeism from work is essential to drive practitioners in their interaction with patients during the RTW process. Our results showed that prognostic factors within the clinical, psycho-social, and social workplace categories are all associated with RTW. Overall, prognostic factors related to high global health well-being, great socioeconomic status, good mental health conditions have been positively associated with RTW outcomes. In the same way, global conditions with less favorable repercussions on workers lives resulted in a delay in time to RTW. Among these were older age, being female, higher pain or disability, depression, higher physical work demands, abuse of smoke and alcohol have shown a strong level of evidence for negative outcomes. Therefore, health and social condition are decisive in RTW outcomes. Our findings confirmed the results of Steensa et al. [38] and Cancelliere et al. [7], from whose research health global status seemed not to influence absenteeism from work. Interestingly, our results also highlighted the crucial role of interventions in the workplace environment since all factors included in this category have strongly influenced RTW. Therefore, occupational physicians (OPs) can play a crucial function also in the context of RTW. In fact, they not only are a fundamental component within the Occupational Safety and Health (OSH) management systems in protecting and improving the health of employees, but these professional figures are also increasingly asked to address issues such as health promotion and occupational rehabilitation in addition to protecting the worker’s health against work-related injuries and occupational diseases [39]. In this regard, OPs have available various resources and operational strategies to facilitate the RTW of a worker with LBP. For example, they could raise workers awareness of occupational risk factors to prevent work-related injuries or disabling conditions and promote best practices in order to foster employee physical and psychological well-being [40]. Moreover, the main task of OPs is to assess the fitness of workers for specific tasks, ensuring a satisfactory fit between person and job, enabling workers to undertake the work they have been selected to perform safely and effectively [41]. However, the best result in terms of efficient and timely RTW can only be achieved by developing a multidisciplinary vocational rehabilitation program that includes the involvement, in addition to OPs, also of other professional figures such as the orthopaedist, physiotherapist, neurosurgeon, nutritionist and psychologist [41, 42]. One of the essential points of this research is the identification of eventual modifiable prognostic factors because these could respond to new interventions targeted at modifying them, improving RTW outcomes. The expectations of recovery, pain intensity, and disability levels, as well as depression, distress, and workplace factors, can be considered among the most important modifiable factors in progressing RTW across health and injury conditions. For example, having hopeful expectations for recovery and RTW was usually correlated with positive RTW outcomes, as shown by evidence from mental health studies. Instead, for people who expected to recover more slowly after an injury, this often happened. This mental condition can lead to a slower recovery and a higher risk of receiving sick leave benefits. But since it is considered a modifiable prognostic factor, it should be early identified. Despite recovery expectations would seem correlated to mental health status, current research has not confirmed this association. Gatchel et al. [36] had shown that neither type nor the degree of psychopathology were significantly predictive of a patient’s ability to return to work successfully. Other important modifiable prognostic factors are those concerning workplace environment conditions. Work accommodation seems to be essential for increasing RTW outcomes, especially across health and injury conditions. Moreover, the opportunity to adopt special accommodations for workers who have suffered an injury/illness or who have acquired a disability condition—and are exposed to specific occupational risk factors—is widely recognized and derives from the need to guarantee a full and satisfactory fit between worker’s health conditions and characteristics of working tasks and activities [43]. Once again, the implementation of an adequate occupational rehabilitation program by the OSH management systems could play a key role in evaluating and identifying the most suitable accommodations to be taken by employers for LBP workers, but also in supporting them with specific and targeted counseling programs and strategies. The present study has several strengths as well as limitations. First, our analysis is restricted to studies with a defined phase of disease, as chronic LBP. In most of the current articles, patients are enrolled in different points of their disease, creating a mixture of the population with workers on sick leave and workers still at work. However, the cut-off for identifying the chronic phase of disease was chosen at 12 weeks based on the median and 75th percentile. Moreover, due to the large number of prognostic factors analyzed, heterogeneous methods in which data are collected have been found, and this may influence the robustness of the results. Therefore, the included studies are still not enough to justify a definitive association between prognostic risk factors and RTW, and further specific studies, with more homogeneous evaluation, should be performed to define these factors definitively.

## Conclusion

This review proved that multidisciplinary interventions should be performed to improve RTW outcomes. A deeper knowledge of the possible causes which delay return to work can provide essential solutions to improved workers condition and productivity annual losses for absenteeism. In particular, high global health well-being, great socioeconomic status, and good mental health conditions are decisive in RTW outcomes. In conclusion, interventions that aim at return-to-work of employees sick-listed with LBP should predominantly focus on psycho-social aspects, health behaviors, and workplace characteristics.
